# Comparative Evaluation of Glidants for Enhancing the Flowability of Poorly Flowing Powdered Materials with Varying Particle Sizes and Morphologies

**DOI:** 10.3390/pharmaceutics18060721

**Published:** 2026-06-11

**Authors:** Daniel Zakowiecki, Peter Edinger, Michael Wagner, Tobias Hess, Dariusz Lipiak, Krzysztof Cal

**Affiliations:** 1 Chemische Fabrik Budenheim KG, Rheinstrasse 27, 55257 Budenheim, Germany; peter.edinger@budenheim.com (P.E.); michael.wagner@budenheim.com (M.W.); tobias.hess@budenheim.com (T.H.); 2 Brenntag Polska sp. z o.o., ul. Migdalowa 4/52, 02-796 Warsaw, Poland; dariusz.lipiak@brenntag.com; 3 Department of Pharmaceutical Technology, Faculty of Pharmacy, Medical University of Gdansk, al. Gen. J. Hallera 107, 80-416 Gdansk, Poland; kcal@wp.pl

**Keywords:** glidants, powder flow, flow enhancement, silicon dioxide, tribasic calcium phosphate, ibuprofen, lecithin, mefenamic acid, metamizole sodium

## Abstract

**Background**: An increasing number of commercially available drug substances and bioactive ingredients are characterized by poor flowability. Inadequate flow properties may lead to material blockage during transport within production lines, as well as the formation of air voids within the bulk. Such phenomena can disrupt the technological process and may even result in batches that fail to meet quality requirements. Therefore, ensuring adequate powder flow is of utmost importance in the manufacture of health-related products. **Methods**: Binary mixtures were prepared using one of four model substances (ibuprofen, metamizole sodium, mefenamic acid, or sunflower lecithin) combined with a glidant (colloidal silica, precipitated silica, or tricalcium phosphate). The glidant content ranged from 0.5 to 10.0% *w*/*w* depending on the model substance, and mixing was carried out for 5–30 min. The resulting binary mixtures were evaluated for flow properties using the angle of repose method, and in selected cases, bulk density was also determined. **Results/Conclusions**: The study demonstrated that powder flow improvement depended not only on the glidant but primarily on the properties of the host material (particle size, shape, and bulk density). Coarser powders such as ibuprofen responded well to low glidant levels, although excessive silicon dioxide caused oversilication. Metamizole sodium showed progressively better flow with increasing particle size and density, and tribasic calcium phosphate further improved performance, particularly with longer mixing times. Very fine or cohesive powders, such as mefenamic acid and sunflower lecithin, showed limited response to silica-based glidants, whereas tribasic calcium phosphate proved more effective and additionally increased bulk density. Overall, no universal glidant strategy was identified; effective flow enhancement requires a tailored approach based on specific powder characteristics.

## 1. Introduction

An increasing number of commercially available drug substances and bioactive ingredients exhibit poor flow properties. Powder flowability is critical for manufacturing operations such as mixing, granulation, capsule filling, and tableting. Inadequate powder flow can lead to blockages during material transport by promoting air pocket formation within the bulk, potentially disrupting production and resulting in batches that fail to meet quality requirements. Enhancing flowability improves process performance, minimizes raw material losses, and ensures uniform dosing in the final product. Therefore, ensuring adequate powder flow is essential for the reliable manufacture of health-related products [1,2,3,4].

Various technological approaches can be employed to improve the flowability of powders. One common method is granulation (either wet or dry), which increases particle size, reduces interparticle cohesion, and decreases the tendency of powders to cake. However, granulation processes extend production cycle times, increase energy consumption, and consequently raise overall production costs.

A practical and beneficial alternative is the use of flow-enhancing aids, commonly referred to as glidants. These substances, applied in relatively small quantities, can convert even poorly flowing powders into materials suitable for demanding direct compression (DC) technology, which is currently the most straightforward and cost-effective method for the production of oral solid dosage forms (OSDFs) [5,6,7,8].

Various glidants are used in the development and production of solid dosage forms, with silicon dioxide being the most commonly used. It has a long history of use in the pharmaceutical industry, is well characterized in pharmacopoeias, and has broad regulatory acceptance. Two types are mainly used in industry, colloidal and precipitated silicas. However, its highly fluffy nature can lead to significant dust generation during processing. It has also been the subject of consumer debate regarding nanomaterials, although no significant toxicological evidence supports related health concerns [9,10,11]. Talc is a clay mineral composed of hydrated magnesium silicate. It is relatively inexpensive and has been traditionally used as a glidant in pharmaceutical and industrial applications to improve powder flow properties. However, it is increasingly perceived as obsolete and is used less frequently, partly due to health and regulatory controversies surrounding inconsistent contamination levels and the potential presence of asbestos in natural talc [12,13,14,15]. Calcium phosphates, such as special grades of fine hydroxyapatite, are not as widely acknowledged as glidants. They are naturally occurring minerals and, in addition to improving flowability, can serve as safe sources of calcium and phosphorus in nutraceuticals or dietary supplements. However, it should be borne in mind that they may form chelates with certain substances present in the formulations [16,17,18].

Glidants in pharmaceutical formulations are used in amounts necessary to achieve the intended technological effect, in accordance with the *quantum satis* principle. Although there are no regulatory requirements specifying exact usage levels, higher concentrations should be scientifically justified, and their impact on the final product’s properties, including mechanical strength and disintegration time, ought to be evaluated. It has also been reported that excessive concentrations of glidants may produce the opposite effect by impairing flowability. Typically, colloidal silica is used at concentrations of 0.1–1% *w*/*w*, whereas talc generally requires higher amounts, usually 1–10% *w*/*w*. Fine tribasic calcium phosphate is effective at concentrations similar to those of silicon dioxide [9,18,19,20,21].

**Figure 1:**
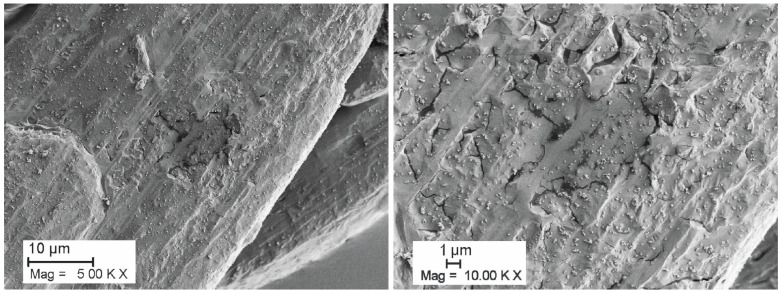
Exemplary SEM images (magnifications 5 kX and 10 kX) showing submicron-sized particles of a glidant uniformly distributed on the surface of crystals of a model drug substance, ibuprofen.

The mechanism of action of glidants is primarily based on reducing friction, as well as cohesion and adhesion between powder particles. Effective glidants consist of very fine particles that deposit onto larger particles of the active substance and excipients, forming a thin surface layer (see Figure 1). This layer smooths the surface and decreases the actual contact area, thereby reducing van der Waals forces and electrostatic interactions between powder particles. Owing to their high specific surface area, glidants can also preferentially adsorb moisture from other particle surfaces, limiting the formation of capillary bridges and preventing agglomeration and caking [22,23,24,25,26].

The aim of the present study was to compare the effects of three commonly applied glidants, i.e., colloidal silicon dioxide, precipitated silica, and fine tribasic calcium phosphate, on the flow properties of selected bioactive substances widely used in medicinal and health-related products. The materials investigated included ibuprofen, metamizole sodium, mefenamic acid, and lecithin, all of which are characterized by poor flowability. Addressing this functional limitation at an early stage of development is essential for the successful production of safe and reliable oral solid dosage forms. The study examined the influence of glidant concentration and mixing time on the flow behaviour of binary mixtures containing various ratios of glidant and model substance. Flow properties were evaluated by measuring the angle of repose, and the powders were classified according to the relative ranking of flow described in pharmacopoeias, including the European Pharmacopoeia (Ph. Eur.) and the United States Pharmacopeia–National Formulary (USP–NF). Values of 25–30 indicate excellent flow properties; 31–35, good flow properties; 36–40, fair flow properties (no aid needed); 41–45, passable flow (the powder may hang up); 46–55, poor flow (the powder must be agitated or vibrated to induce flow); 56–65, very poor flow; and above 66, very, very poor flow. Additionally, the effect of glidants on bulk density was analyzed, as this parameter may influence both powder flowability and the size of the final dosage forms [27,28,29].

## 2. Materials and Methods

**Figure 2:**
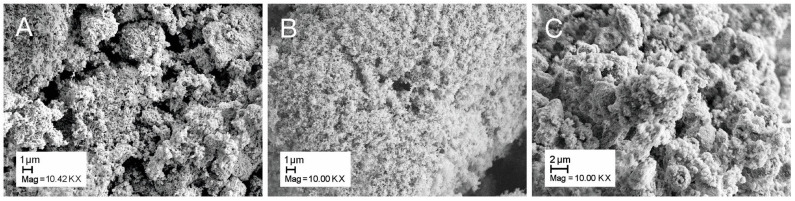
SEM images (×10,000) of tribasic calcium phosphate, TCP (**A**); colloidal silicon dioxide, CSD (**B**); precipitated silica, PSD (**C**).

Glidants: colloidal silicon dioxide (**CSD**), Aerosil^®^ 200 from Evonik Operations GmbH (Essen, Germany), with a bulk density of about 30 g/mL and a specific surface area of approximately 200 m^2^/g; precipitated silicon dioxide (**PSD**), Syloid^®^ 244 FP from Grace GmbH & Co. KG (Worms, Germany), exhibiting a bulk density near 70 g/mL and a surface area of roughly 350 m^2^/g; tribasic calcium phosphate (**TCP**), PharSQ^®^ Flow T 200 from Chemische Fabrik Budenheim KG (Budenheim, Germany), characterized by a bulk density of about 200 g/mL and a specific surface area close to 66 m^2^/g [30,31,32,33,34]. Examples of scanning electron microscope (SEM) images of the glidants used in this study, taken at a magnification of 10,000×, are presented in Figure 2.

Model bioactive substances: sunflower lecithin (**LEC**) powder from National Lecithin (Hale Cheshire, UK); mefenamic acid (**MA**) from Yung Zip Chemical Ind Co., Ltd. (Dajia, Taiwan); ibuprofen 50 (**IBU_50**) from BASF ChemTrade GmbH (Burgbernheim, Germany); ibuprofen SN (**IBU_SN**) from Strides Shasun Ltd. (Puducherry, India); metamizole sodium monohydrate (**MSM 1**) from Sofarimex Indústria Química e Farmacêutica, S.A. (Lisboa, Portugal); metamizole sodium monohydrate (**MSM 2**) from Shandong Xinhua Pharmaceutical Co., Ltd. (Zibo, China); metamizole sodium monohydrate (**MSM 3**) from Vani Pharma Labs Ltd. (Hyderabad, India).

### 2.1. Preparation of Binary Mixtures Containing Ibuprofen, Metamizole Sodium Monohydrate, and Mefenamic Acid

Each model drug substance was mixed individually with the selected glidant at concentrations of 0.5, 1.0, 1.5, and 5.0% *w*/*w* (relative to the total mixture mass). Since some glidants, particularly CSD and PSD, tended to agglomerate and form lumps, all powder components were manually passed through a 1 mm sieve before mixing to ensure a consistent preparation procedure. After sieving, the components were accurately weighed to obtain approximately 300 g of the powder mixture and transferred to a 500 mL plastic mixing container, such that the powder volume occupied 50% or less of the container volume. The powder mixtures were blended using a Turbula^®^ mixer (Willy A. Bachofen AG, Muttenz, Switzerland) at 32 rpm for 5, 15, or 30 min.

For binary mixtures of MA and TCP, additional samples containing 7.5 and 10.0% *w*/*w* TCP were prepared using the same procedure.

### 2.2. Preparation of Binary Mixtures Containing Sunflower Lecithin Powder

Sunflower lecithin powder (LEC) used in this study was sticky and exhibited a strong tendency to agglomerate, forming relatively spherical lumps. In addition, its high moisture uptake further promoted agglomeration. Therefore, lecithin and each glidant were manually sieved separately through a 1 mm mesh sieve prior to mixing to ensure a consistent preparation procedure.

Binary mixtures of LEC with the selected glidant were prepared at concentrations of 1.0, 2.5, 5.0, 7.5, and 10.0% *w*/*w* (relative to the total mixture mass). After sieving, the components were accurately weighed to obtain approximately 300 g of the powder mixture and transferred to a 500 mL plastic mixing container, such that the powder volume occupied 50% or less of the container volume. The powder mixtures were blended using a Turbula^®^ mixer (Willy A. Bachofen AG, Muttenz, Switzerland) at 32 rpm for 2 min. The mixtures were then dried at 50 °C for 30 min and passed through a 0.8 mm mesh sieve. Subsequently, the powders were returned to the mixing container and blended at 32 rpm to achieve total mixing times of 5, 15, and 30 min.

### 2.3. Flowability and Bulk Density Measurements

Immediately after preparation, the binary powder mixtures were evaluated for their flow properties using the angle of repose (AoR) method, following the procedure described in Ph. Eur. 11.5, chapter 2.9.36 “Powder flow”. In addition, untapped bulk density was measured according to Ph. Eur. 11.5, chapter 2.9.34 “Bulk density of powders”.

### 2.4. Scanning Electron Microscopy (SEM) and Optical Microscopy

Scanning electron microscopy (SEM) was performed using a scanning electron microscope (Carl Zeiss Microscopy GmbH, Oberkochen, Germany) at an accelerating voltage of 2 kV, equipped with a secondary electron (SE) detector. The aperture size was set to 30 µm. Micrographs were acquired at magnifications ranging from 200× to 10,000× under a vacuum of <5.0 × 10^−5^ mbar.

Optical microscopic analysis was performed using a digital microscope (Keyence^®^ VHX-5000, Keyence Corporation, Osaka, Japan). The lecithin sample was dispersed in dimethicone oil on a glass microscope slide and observed at magnifications ranging from 300× to 1000×.

### 2.5. Particle Size Distribution and Shape Analysis

Particle size distribution (PSD) analysis was conducted using dynamic image analysis on a CAMSIZER X2 apparatus (Retsch Technology GmbH, Haan, Germany) at 2.0 bar pressure using air dispersion with an “X-Jet” module. The results were calculated as the equivalent particle diameter, corresponding to the diameter of a circle with equivalent area, and are presented as D10, D50, and D90 values. From these measurements, additional particle shape parameters were acquired, including aspect ratio (AR) and sphericity (SPHT).

## 3. Results and Discussion

### 3.1. Ibuprofen

**Figure 3:**
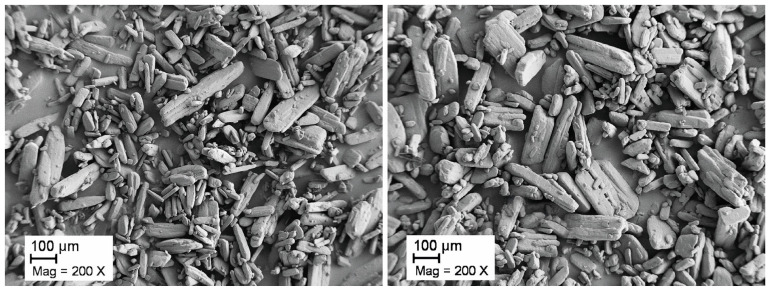
SEM images of ibuprofen crystals at 200× magnification: IBU_50 (**left**) and IBU_SN (**right**).

In this study, the influence of glidants on the flow characteristics of IBU from two different manufacturers was investigated. Although the particle shapes were similar (see Figure 3), IBU_50 consisted of smaller particles (D10 ~ 20 µm, D50 ~ 48 µm, D90 ~ 106 µm), whereas IBU_SN contained larger ones (D10 ~ 27 µm, D50 ~ 73 µm, D90 ~ 156 µm). The grades also differed in bulk density, measuring approximately 320 g/L for IBU_50 and 395 g/L for IBU_SN. Neither material flowed freely, and the measured angle of repose exceeded 70°, which, according to Ph. Eur. and USP–NF standards, corresponds to very, very poor flowability. The detailed characterization of the IBU samples is provided in the Supplementary Materials.

**Figure 4:**
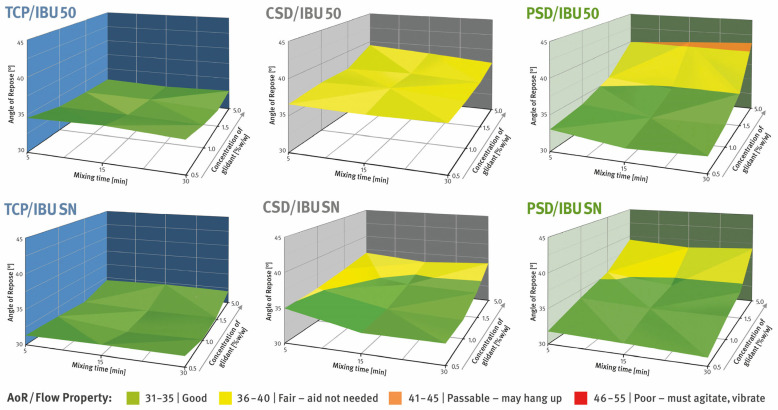
The angle of repose (AoR) of binary mixtures of ibuprofen (IBU_50 and IBU_SN), with concentrations of glidants (TCP, CSD, and PSD) ranging from 0.5% to 5.0% *w*/*w*, mixed for 5 to 30 min (mean of *n* = 3). The arrow indicates increasing glidant concentration. Detailed data, together with a statistical analysis of the results, are provided in the Supplementary Materials.

Despite the unfavorable elongated, columnar crystal morphology, both IBU types exhibited high sensitivity to the presence of glidants (see Figure 4). In all cases, a substantial improvement in powder flowability was observed. However, IBU_SN showed greater responsiveness to the addition of glidants, as demonstrated by lower values of the angle of repose, indicating enhanced flow properties. This behaviour was likely related to its larger particle size and higher bulk density. Bigger particles had a lower specific surface area, which reduced the relative contribution of interparticle cohesive forces, such as van der Waals interactions. As a result, cohesion was diminished, facilitating better flow and increasing the effectiveness of glidants. Similar phenomena have been reported elsewhere [35,36,37].

A comparison of individual glidants showed that TCP was highly effective in improving the flowability of both IBU types, bringing it from very, very poor to good according to Ph. Eur./USP–NF. Unlike typical glidants, it did not exhibit adverse effects related to overloading, which means that increasing its concentration or extending the mixing time had no unfavorable impact on powder flow. In contrast, both silicas demonstrated a deterioration in performance at higher glidant concentrations. As their levels increased, the flowability of the binary mixtures with IBU worsened, with a more pronounced effect observed for PSD. Although PSD performed slightly better than CSD at lower concentrations, further additions markedly impaired the flowability of the binary mixtures. This phenomenon, known as oversilication, has been previously reported in the literature and is caused by excess silica glidant loading beyond the optimal surface coverage of the host particles, defined as the level at which a uniform layer of silica sufficiently coats the particle surfaces without forming agglomerates [19,26].

### 3.2. Metamizole Sodium Monohydrate

**Figure 5:**
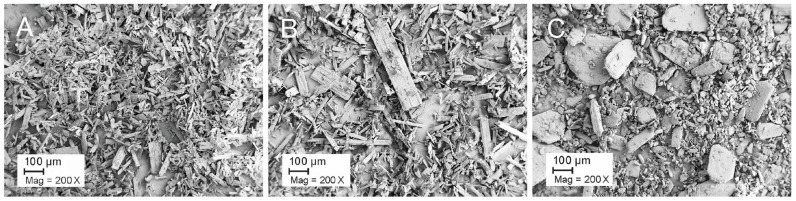
SEM images of metamizole sodium monohydrate crystals at 200× magnification: MSM 1 (**A**), MSM 2 (**B**), and MSM 3 (**C**).

**Figure 6:**
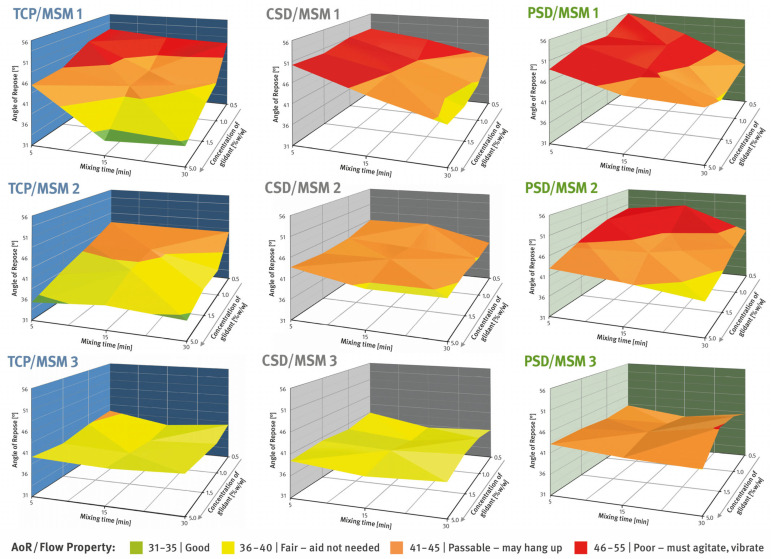
The angle of repose (AoR) of binary mixtures of MSM, with concentrations of glidants (TCP, CSD, and PSD) ranging from 0.5% to 5.0% *w*/*w*, mixed for 5 to 30 min (mean of *n* = 3). The arrow indicates increasing glidant concentration. Detailed data, together with a statistical analysis of the results, are provided in the Supplementary Materials.

The metamizole sodium monohydrate samples examined in this study differed in particle shape, particle size, and bulk density (Figure 5). MSM 1, with a bulk density (BD) of approximately 209 g/mL, consisted predominantly of acicular to lath-like crystals, with particle sizes of D10 ≈ 10 µm, D50 ≈ 24 µm, and D90 ≈ 58 µm. MSM 2, with a slightly higher BD of approximately 245 g/mL, exhibited a similar particle shape. Although a few relatively large crystals were observed (Figure 5A), the particle size distribution was D10 ≈ 11 µm, D50 ≈ 27 µm, and D90 ≈ 54 µm. In contrast, MSM 3 exhibited a different morphology, characterized by blocky to plate-like particles with angular edges. The particle size distribution was D10 ≈ 18 µm, D50 ≈ 64 µm, and D90 ≈ 256 µm, and the BD was approximately 482 g/mL. These properties affected the flow characteristics of MSM powders, as well as their sensitivity to the presence of a glidant (see Figure 6). The AoR measured for the neat drug substances was 59.9° for MSM 1 and 56.8° for MSM 2, which, according to Ph. Eur./USP–NF, correspond to very poor flow properties, and 52.7° for MSM 3, corresponding to poor flowability. The detailed characterization of the MSM samples is provided in the Supplementary Materials.

MSM 1, with the smallest particles and lowest bulk density, showed the poorest flowability. However, this drug substance was sensitive to the presence of a glidant. The most pronounced effect was observed for binary mixtures with TCP, where flow properties improved markedly with increasing glidant concentration and mixing time. In the case of CSD, the material was insensitive to glidant concentration, whereas longer mixing noticeably enhanced flowability. When comparing both silica types, CSD showed slightly better performance. PSD, particularly at shorter mixing times, exhibited only minor improvement with increasing glidant levels.

MSM 2, with slightly larger particles and higher bulk density, exhibited somewhat better initial flowability and, similarly to MSM 1, remained sensitive to the presence of a glidant. For TCP, a measurable improvement was observed with increasing glidant concentration; however, extended mixing had no effect on the measured AoR values. In contrast, for both silicas, higher glidant levels and longer mixing enhanced flow behaviour, with CSD again showing slightly better performance than PSD.

MSM 3, characterized by the largest, irregular crystals and the highest bulk density, exhibited better initial flowability than the other two MSM types. A small addition of glidant significantly improved flowability, while further increases in concentration or mixing time did not yield additional advantages. TCP and CSD showed comparable effects on the flow behaviour of the binary mixtures, whereas PSD performed slightly worse.

The results obtained showed that the same pharmaceutical substance can differ significantly in its physical characteristics, including powder flow behaviour, depending on its source or manufacturer [38,39]. Consequently, the addition of glidants may result in variable effects on flowability. Several factors contribute to these differences, including the type of glidant, its concentration, and the mixing time with poorly flowing powders. Equally important are the intrinsic properties of the poorly flowing substance, such as particle size, shape, morphology, and density [15,35,36]. Such variations can be critical in drug formulation technology. Manufacturers often rely on multiple suppliers of pharmaceutical substances, and differences between them can influence the manufacturing process. For example, in technologies such as direct compression or continuous manufacturing, poor flowability can result in products exhibiting a lack of uniformity in dosage units and may even lead to the rejection of an entire production batch [2,6,40,41].

Typically, glidants in pharmaceutical preparations are used in very small quantities, conventionally not exceeding 2% *w*/*w*. However, the present experiments indicate that, in some cases, higher concentrations can positively impact the flowability of powder mixtures. Notably, even with colloidal silica, no oversilication effect was observed in the examined MSM types when the concentration was increased or the mixing time was extended [9,24,42,43].

### 3.3. Mefenamic Acid

**Figure 7:**
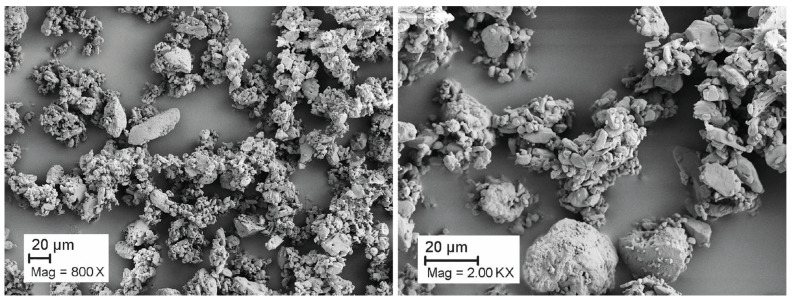
SEM images of mefenamic acid crystals at 800× (**left**) and 2000× (**right**) magnification.

The investigated mefenamic acid powder exhibited extremely poor flowability, as indicated by an angle of repose exceeding 66°. This unfavorable property was attributed to the very fine particle size, with a particle size distribution of D10 ≈ 5 µm, D50 ≈ 13 µm, and D90 ≈ 44 µm, as well as a very low bulk density of approximately 193 g/L. The majority of the crystals displayed a flake-like morphology and formed loose agglomerates, which likely disintegrated into primary particles during processing. Additionally, larger, irregularly shaped particles were observed (see Figure 7). The detailed characterization of the MA sample is provided in the Supplementary Materials.

**Figure 8:**
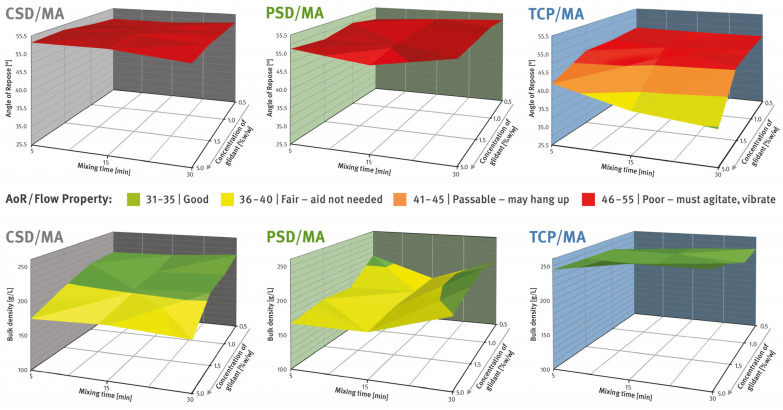
The angle of repose (AoR) and bulk density of binary mixtures of MA, with concentrations of glidants (TCP, CSD, and PSD) ranging from 0.5% to 5.0% *w*/*w*, mixed for 5 to 30 min (mean of *n* = 3). The arrow indicates increasing glidant concentration. Detailed data, together with a statistical analysis of the results, are provided in the Supplementary Materials.

**Figure 9:**
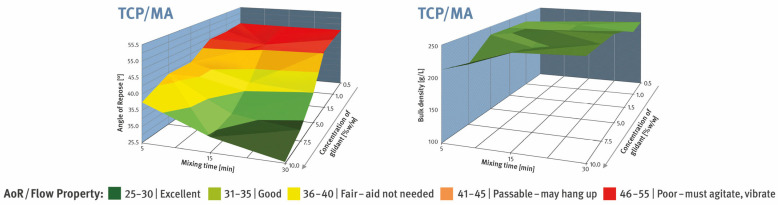
The angle of repose (AoR) and bulk density of binary mixtures of MA with TCP which concentration ranged from 0.5% to 10.0% *w*/*w*, mixed for 5 to 30 min (mean of *n* = 3). The arrow indicates increasing glidant concentration. Detailed data, together with a statistical analysis of the results, are provided in the Supplementary Materials.

For powders composed of very fine particles, characterized by high specific surface area and elevated surface energy, the use of glidants often does not significantly improve flowability. In such particulate systems, interparticle cohesive forces dominate over gravitational forces, severely inhibiting flow. Under these conditions, glidants provide little to no benefit, as their ability to reduce effective contact forces is insufficient [22,44,45,46]. The results presented here partially confirm these observations—see Figure 8. Regardless of concentration and mixing time, both silicas formed binary mixtures with MA that exhibited poor flow. In contrast, systems containing TCP showed progressive improvement as the glidant concentration increased and mixing time was extended. Thus, the study was continued using higher TCP levels, ranging from 5 to 10% *w*/*w*. The corresponding results are presented in Figure 9 and demonstrate a further enhancement in flow behaviour. The measured angle of repose (AoR) values reached as low as 25–30°, which, according to Ph. Eur./USP–NF, corresponds to excellent flow properties.

The tests additionally comprised an assessment of the impact of glidants on the bulk density of binary mixtures with mefenamic acid (see Figure 8). It was observed that, in the case of both silicon dioxides, the bulk densities of the powders were relatively low and slightly decreased further with increasing glidant concentration. Such an increase in bed porosity following the addition of a glidant occurs in non-optimal systems, where the glidant is not uniformly distributed across the surface of host particles. In these cases, surface coverage is incomplete and the glidant tends to agglomerate, forming a porous network that disrupts efficient particle packing. This effect is not systematically observed but depends on the properties of the host powder, such as particle size and shape, as well as on the mixing conditions and the energy required to achieve adequate dispersion of the glidant [19,23,47,48].

In binary mixtures of MA with TCP, the opposite effect was observed. The bulk density increased slightly with rising glidant concentration, which, beyond a certain point, was accompanied by a gradual decrease in the angle of repose, eventually reaching values indicative of good flow properties. This behaviour can be attributed to the higher density of TCP. The heavier particles promoted rearrangement of the host particles, leading to more efficient packing and, consequently, reduced bed porosity. A higher density means that the powder occupies a smaller volume, which can directly impact the size of the final dosage form. Smaller tablets or hard capsules are generally better accepted by patients and may positively influence patient compliance [49,50,51,52].

### 3.4. Sunflower Lecithin

**Figure 10:**
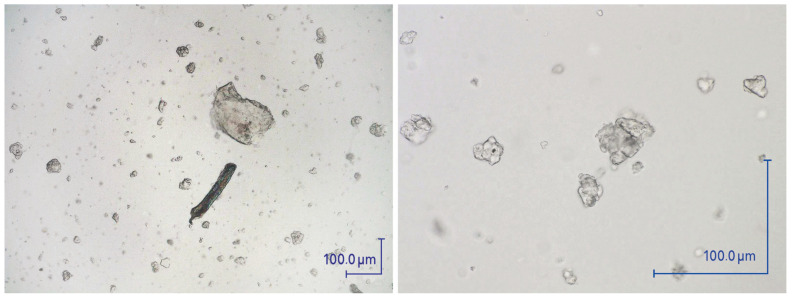
Microscopic images of sunflower lecithin powder at 300× (**left**) and 1000× (**right**) magnification.

The sunflower lecithin powder examined in the present study (see Figure 10) exhibited relatively high viscosity, causing it to adhere strongly to the surfaces of measuring devices and significantly hindering the measurement process. This behaviour can be attributed to the specific physical properties of powdered sunflower lecithin, which arise from its lipid–amphiphilic nature and promote interparticle lipid interactions. Additionally, the presence of hydrophilic groups (phosphate and choline) contributes to a strong tendency to absorb moisture and agglomerate, leading to the formation of relatively large, soft lecithin agglomerates [53,54,55]. In combination with the relatively small particle sizes (D10 ≈ 7 µm, D50 ≈ 25 µm, and D90 ≈ 143 µm), these characteristics resulted in poor powder flowability, as indicated by AoR values of approximately 49°. The detailed characterization of the LEC sample is provided in the Supplementary Materials.

**Figure 11:**
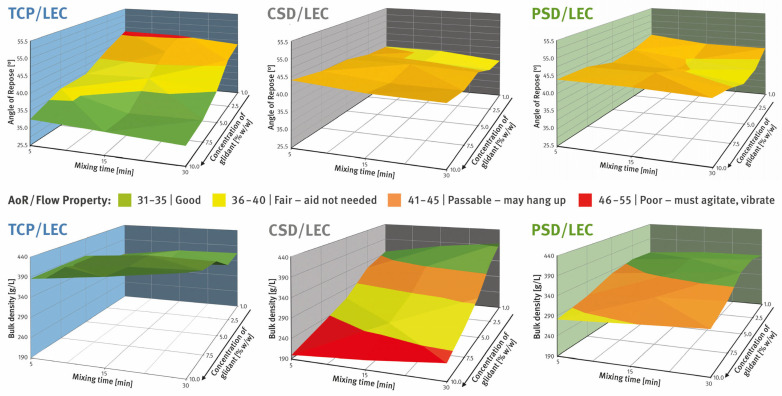
The angle of repose (AoR) and bulk density of binary mixtures of LEC, with concentrations of glidants (TCP, CSD, and PSD) ranging from 1.0% to 10.0% *w*/*w*, mixed for 5 to 30 min (mean of *n* = 3). The arrow indicates increasing glidant concentration. Detailed data, together with a statistical analysis of the results, are provided in the Supplementary Materials.

The tested glidants improved the flow properties of the binary mixtures with lecithin in different ways (see Figure 11). Both grades of silica enhanced flowability from a passable to a fair level across the tested concentrations and mixing times. CSD showed a slight improvement with longer mixing time; however, higher concentrations somewhat worsened the flowability of the tested mixtures. For PCD, optimal flowability enhancement occurred at concentrations of 2.5–5% *w*/*w* and a 15–30 min mixing time. TCP behaved differently, and at higher concentrations it markedly improved the flowability of binary mixtures with lecithin to a good level, with further enhancement observed upon prolonged mixing time.

The glidants also affected bulk density. TCP slightly increased the density of the binary mixtures with lecithin, while both grades of silica decreased it, especially CSD. These effects may be attributed to differences in particle morphology (see Figure 2) and true density, ~3.14 g/cm^3^ for TCP versus ~2.2 g/cm^3^ for SiO_2_ [9]. Despite its high porosity, TCP particles were heavier and better separated lecithin particles, limiting agglomeration and improving flowability.

It is also worth noting that lecithin powder is hygroscopic; moisture uptake can increase interparticle cohesion, leading to clumping and reduced flowability. A higher concentration of a highly porous glidant with an elevated specific surface area can actively absorb moisture from the environment and mitigate this adverse effect [56,57,58,59].

## 4. Conclusions

The aim of this study was to evaluate the effect of three commonly used glidants in the pharmaceutical, nutraceutical, and dietary supplement industries on the flow properties of selected model substances with poor flowability: ibuprofen, metamizole sodium, mefenamic acid, and sunflower lecithin. The selection and number of materials were constrained by market availability. For ibuprofen and metamizole sodium, more product types were investigated, which allowed evaluation of the influence of material variability within a single drug substance type.

The investigated glidants differed in chemical nature, particle morphology, bulk density, and specific surface area. Silicon dioxide–based glidants are insoluble in water and most inorganic solvents. Tribasic calcium phosphate, while also insoluble in water, dissolves in dilute acids and therefore in the acidic environment of the stomach under fasting conditions. In addition, silica creates an acidic microenvironment, whereas tribasic calcium phosphate remains neutral, which may make it more suitable for acid-sensitive drug substances.

The results demonstrate that improvements in powder flowability depend not only on glidant properties but also on the characteristics of the host material, including particle size, shape, and bulk density. For materials with relatively larger crystals, including ibuprofen, even low glidant concentrations significantly enhanced flow despite unfavourable particle morphology. However, both silicon dioxide types exhibited an oversilication effect at higher concentrations.

For metamizole sodium, glidant performance was strongly influenced by particle size and density. Effectiveness increased alongside both parameters of the host powder. No oversilication was observed. In contrast, higher glidant levels improved flow, particularly in samples composed of smaller crystals. Prolonged mixing time further enhanced flowability, with the most pronounced effect seen for tribasic calcium phosphate.

More challenging materials, i.e., very fine powders (mefenamic acid) and sticky, highly hygroscopic substances (lecithin), showed only limited improvement when processed with silica-based glidants. Under these conditions, tribasic calcium phosphate proved more effective, especially at concentrations exceeding those typically applied in pharmaceutical formulations. Its addition also increased the bulk density of the mixtures, which may improve manufacturing efficiency and reduce final dosage form size.

Overall, the findings indicate that no single glidant or set of process parameters can be universally recommended. Each poorly flowing powder requires an individualized approach. Accordingly, studies such as those presented in this paper are particularly valuable during early-stage development of health-related products containing difficult-to-handle ingredients.

## Data Availability

The original contributions presented in this study are included in the article and Supplementary Material. Further inquiries can be directed to the corresponding author.

## Supplementary Materials

The following supporting information can be downloaded at https://www.mdpi.com/article/10.3390/pharmaceutics18060721/s1.
