# Supplementary material for: Comparative Evaluation of Glidants for Enhancing the Flowability of Poorly Flowing Powdered Materials with Varying Particle Sizes and Morphologies
Source: Pharmaceutics. 2026 Jun 11;18(6):721. doi: 10.3390/pharmaceutics18060721 (PMC13306053; doi:10.3390/pharmaceutics18060721)
Supplement: Supplementary file 1 [file pharmaceutics-18-00721-s001.zip › pharmaceutics-4335380-supplementary.pdf]

Supplementary Materials

Comparative Evaluation of Glidants for Enhancing the Flowability of Poorly Flowing Powdered Materials with Varying Particle Sizes and Morphologies

Daniel Zakowiecki, Peter Edinger, Michael Wagner, Tobias Hess, Dariusz Lipiak and Krzysztof Cal

S1. Ibuprofen

Table S1. Characteristics of the ibuprofen samples used in the study.

Sample	D10 [µm]	D50 [µm]	D90 [µm]	Sphericity	Aspect ratio	BD [g/L]	AoR [°]
**IBU_50**	20 ± 0.3	48 ± 0.4	106 ± 3.7	0.728 ± 0.016	0.642 ± 0.013	320 ± 3.7	> 70
**IBU_SN**	27 ± 0.5	73 ± 0.9	156 ± 6.2	0.741 ± 0.007	0.643 ± 0.006	395 ± 5.2	> 70

Table S2. The angle of repose (AoR) of binary mixtures of ibuprofen IBU_50 with concentrations of glidants (TCP, CSD, and PSD) ranging from 0.5% to 5.0% w/w, mixed for 5 to 30 minutes (mean of n = 3).

		**IBU_50**		
		**TCP**	**CSD**	**PSD**
Glidant Level [%]	Mixing time [min]	AoR [°]	AoR [°]	AoR [°]
5.0	5	33.1 ± 0.7	39.4 ± 0.3	39.9 ± 0.7
5.0	15	32.7 ± 0.2	38.6 ± 0.8	40.6 ± 0.9
5.0	30	32.9 ± 0.2	37.8 ± 0.3	41.1 ± 0.7
1.5	5	33.2 ± 0.2	37.2 ± 0.2	35.6 ± 0.3
1.5	15	33.0 ± 0.4	37.3 ± 0.3	34.7 ± 0.5
1.5	30	32.4 ± 0.7	36.8 ± 0.3	35.0 ± 0.8
1.0	5	33.5 ± 1.1	37.2 ± 0.7	33.8 ± 0.3
1.0	15	33.4 ± 0.2	37.3 ± 0.3	32.9 ± 0.3
1.0	30	33.1 ± 1.0	36.5 ± 0.5	33.1 ± 0.4
0.5	5	34.6 ± 0.3	36.6 ± 0.4	33.0 ± 0.5
0.5	15	34.7 ± 1.0	36.3 ± 0.9	31.8 ± 0.6
0.5	30	34.2 ± 0.5	36.4 ± 0.5	32.1 ± 0.3

Table S3. Three-way ANOVA showing the effects of glidant type and glidant concentration on the angle of repose (AoR) of IBU_50 mixtures, along with Tukey's post hoc comparisons among glidants.

Source of variation	df	F-value	p-value	Significance
Glidant type (TCP, CSD, PSD)	2	4.52×10^{26}	< 0.0001	Significant
Glidant concentration	3	6.18×10^{26}	< 0.0001	Significant
Mixing time	2	1.27×10^{26}	< 0.0001	Significant
Glidant type × concentration	6	2.73×10^{25}	< 0.0001	Significant
Glidant type × mixing time	4	1.95×10^{25}	< 0.0001	Significant
Glidant concentration × mixing time	6	2.11×10^{25}	< 0.0001	Significant
Glidant type × concentration× mixing time	12	1.64×10^{25}	< 0.0001	Significant

Comparison	Mean difference (°)	p-value (adjusted)	Significance
TCP vs. CSD	−3.52	< 0.001	Significant

TCP vs. PSD	−2.18	0.014	Significant
CSD vs. PSD	1.34	0.121	Not significant

Summary: *Three-way ANOVA revealed that glidant type, concentration, and mixing time significantly affected AoR of IBU_50 mixtures ($p < 0.001$), with significant interaction effects indicating that the influence of concentration and mixing time depends on the glidant type; post-hoc analysis showed that TCP differs significantly from both CSD and PSD, while CSD and PSD do not differ significantly.*

Table S4. The angle of repose (AoR) of binary mixtures of ibuprofen IBU_SN with concentrations of glidants (TCP, CSD, and PSD) ranging from 0.5% to 5.0% w/w, mixed for 5 to 30 minutes (mean of n = 3).

		**Ibuprofen_SN**		
		**TCP**	**CSD**	**PSD**
Glidant Level [%]	Mixing time [min]	AoR [°]	AoR [°]	AoR [°]
5.0	5	32.0 ± 0.2	36.9 ± 1.0	39.3 ± 0.4
5.0	15	32.2 ± 0.3	36.0 ± 0.2	38.6 ± 0.3
5.0	30	32.0 ± 0.2	36.6 ± 0.2	39.1 ± 0.2
1.5	5	30.6 ± 0.3	36.0 ± 1.1	35.5 ± 0.4
1.5	15	30.3 ± 0.2	34.3 ± 0.6	34.1 ± 0.4
1.5	30	30.4 ± 0.2	34.1 ± 0.6	34.1 ± 0.4
1.0	5	31.1 ± 0.5	35.2 ± 0.4	33.8 ± 0.7
1.0	15	29.7 ± 0.1	33.1 ± 0.2	33.3 ± 0.8
1.0	30	30.2 ± 0.4	32.7 ± 0.2	32.8 ± 0.5
0.5	5	31.4 ± 0.9	34.9 ± 0.2	31.8 ± 0.3
0.5	15	31.5 ± 0.1	32.8 ± 0.2	31.5 ± 0.2
0.5	30	31.4 ± 0.3	32.8 ± 0.2	31.1 ± 0.4

Table S5. Three-way ANOVA showing the effects of glidant type and glidant concentration on the angle of repose (AoR) of IBU_SN mixtures, along with Tukey's post hoc *comparisons among glidants.*

**Source of variation**	**df**	**F-value**	**p-value**	**Significance**
Glidant type (TCP, CSD, PSD)	2	5.11×10^{26}	< 0.0001	Significant
Glidant concentration	3	7.42×10^{26}	< 0.0001	Significant
Mixing time	2	9.85×10^{25}	< 0.0001	Significant
Glidant type × concentration	6	3.08×10^{25}	< 0.0001	Significant
Glidant type × mixing time	4	2.14×10^{25}	< 0.0001	Significant
Glidant concentration × mixing time	6	1.96×10^{25}	< 0.0001	Significant
Glidant type × concentration× mixing time	12	1.52×10^{25}	< 0.0001	Significant

Comparison	Mean difference (°)	p-value (adjusted)	Significance
TCP vs. CSD	−3.58	< 0.001	Significant
TCP vs. PSD	−2.74	0.003	Significant
CSD vs. PSD	0.84	0.238	Not significant

Summary: *Three-way ANOVA confirmed that glidant type, concentration, and mixing time significantly affected AoR of IBU_SN mixtures ($p < 0.001$), with significant interaction effects indicating that the influence of concentration and mixing time depends on the glidant type. Post hoc analysis demonstrated that TCP exhibits significantly lower AoR than both CSD and PSD, while no significant difference was observed between CSD and PSD.*

S2. Metamizole sodium monohydrate

Table S6. Characteristics of the metamizole sodium monohydrate samples used in the study.

Sample	D10 [µm]	D50 [µm]	D90 [µm]	Sphericity	Aspect ratio	BD [g/L]	AoR [°]
**MSM 1**	9.8 ± 0.2	24.4 ± 0.9	57.6 ± 2.3	0.657 ± 0.003	0.565 ± 0.004	209 ± 6.3	59.9 ± 2.6
**MSM 2**	11.1 ± 0.3	26.8 ± 0.5	53.5 ± 1.7	0.653 ± 0.004	0.560 ± 0.006	245 ± 3.1	56.8 ± 1.9
**MSM 3**	17.7 ± 0.8	64.4 ± 5.0	256.3± 14.4	0.742 ± 0.007	0.632 ± 0.005	482 ± 15.5	52.7 ± 0.8

Table S7. The angle of repose (AoR) of binary mixtures of metamizole sodium monohydrate MSM 1 with concentrations of glidants (TCP, CSD, and PSD) ranging from 0.5% to 5.0% w/w, mixed for 5 to 30 minutes (mean of n = 3).

		**MSM 1**		
		**TCP**	**CSD**	**PSD**
Glidant Level [%]	Mixing time [min]	AoR [°]	AoR [°]	AoR [°]
0.5	5	51.3 ± 0.3	51.2 ± 0.8	55.9 ± 1.3
0.5	15	49.7 ± 0.5	48.9 ± 0.8	50.5 ± 0.7
0.5	30	49.1 ± 0.3	45.0 ± 0.6	42.3 ± 1.3
1.0	5	50.0 ± 0.3	51.2 ± 0.6	51.4 ± 1.1
1.0	15	43.2 ± 0.8	46.7 ± 0.9	50.0 ± 1.5
1.0	30	40.9 ± 1.0	40.4 ± 1.2	41.5 ± 1.5
1.5	5	46.0 ± 1.1	49.7 ± 0.5	51.6 ± 1.4
1.5	15	42.8 ± 1.0	45.9 ± 0.3	46.8 ± 0.6
1.5	30	38.6 ± 1.5	39.3 ± 0.8	40.6 ± 0.7
5.0	5	45.4 ± 0.9	50.2 ± 1.3	48.9 ± 1.0
5.0	15	34.4 ± 0.7	44.8 ±0.7	44.4 ± 1.0
5.0	30	35.5 ± 1.2	40.2 ± 0.7	43.6 ± 0.9

Table S8. Three-way ANOVA showing the effects of glidant type and glidant concentration on the angle of repose (AoR) of MSM 1 mixtures, along with Tukey's post hoc *comparisons among glidants.*

Source of variation	df	F-value	p-value	Significance
Glidant type (TCP, CSD, PSD)	2	7.87×10^{26}	< 0.0001	Significant
Glidant concentration	3	1.44×10^{27}	< 0.0001	Significant
Mixing time	2	5.18×10^{27}	< 0.0001	Significant
Glidant type × concentration	6	2.43×10^{26}	< 0.0001	Significant
Glidant type × mixing time	4	1.31×10^{26}	< 0.0001	Significant
Glidant concentration × mixing time	6	9.14×10^{25}	< 0.0001	Significant
Glidant type × concentration× mixing time	12	8.47×10^{25}	< 0.0001	Significant

Comparison	Mean difference (°)	p-value (adjusted)	Significance
TCP vs. CSD	−2.22	0.132	Not significant
TCP vs. PSD	−3.38	0.0104	Significant
CSD vs. PSD	−1.17	0.565	Not significant

Summary: *Three-way ANOVA showed that glidant type, concentration, and mixing time significantly influenced AoR of MSM 1 mixtures (p < 0.001), with significant interaction effects indicating that the impact of concentration and mixing time on powder flowability depends on the glidant type. Post hoc analysis showed that TCP differs significantly from PSD, while differences between TCP and CSD, CSD and PSD are not significant.*

Table S9. The angle of repose (AoR) of binary mixtures of metamizole sodium monohydrate MSM 2 with concentrations of glidants (TCP, CSD, and PSD) ranging from 0.5% to 5.0% w/w, mixed for 5 to 30 minutes (mean of n = 3).

		**MSM 2**		
		**TCP**	**CSD**	**PSD**
Glidant Level [%]	Mixing time [min]	AoR [°]	AoR [°]	AoR [°]
0.5	5	45.6 ± 0.6	45.9 ± 0.3	49.7 ± 1.3
0.5	15	45.2 ± 0.3	45.9 ± 0.3	51.7 ± 0.3
0.5	30	45.0 ± 0.6	41.6 ± 0.7	44.9 ± 1.1
1.0	5	41.9 ± 1.2	43.6 ± 1.2	48.9 ± 1.7
1.0	15	41.9 ± 1.5	42.4 ± 1.3	45.7 ± 1.3
1.0	30	37.7 ± 1.5	40.9 ± 0.4	43.3 ± 1.4
1.5	5	38.9 ± 1.5	43.0 ± 0.7	46.4 ± 1.0
1.5	15	40.5 ± 0.8	43.2 ± 1.2	44.4 ± 0.6
1.5	30	37.7 ± 0.4	41.3 ± 1.7	40.7 ± 1.0
5.0	5	35.2 ± 0.9	43.6 ± 0.9	42.8 ± 0.7
5.0	15	36.7 ± 1.6	40.5 ± 0.4	41.3 ± 0.9
5.0	30	35.5 ± 1.2	40.5 ± 0.4	39.1 ± 1.3

Table S10. Three-way ANOVA showing the effects of glidant type and glidant concentration on the angle of repose (AoR) of MSM 2 mixtures, along with Tukey's post hoc *comparisons among glidants.*

**Source of variation**	**df**	**F-value**	**p-value**	**Significance**
Glidant type (TCP, CSD, PSD)	2	2.32×10^{27}	< 0.0001	Significant
Glidant concentration	3	2.38×10^{27}	< 0.0001	Significant
Mixing time	2	1.14×10^{27}	< 0.0001	Significant
Glidant type × concentration	6	2.25×10^{26}	< 0.0001	Significant
Glidant type × mixing time	4	1.19×10^{26}	< 0.0001	Significant
Glidant concentration × mixing time	6	4.61×10^{25}	< 0.0001	Significant
Glidant type × concentration× mixing time	12	4.78×10^{25}	< 0.0001	Significant

Comparison	Mean difference (°)	p-value (adjusted)	Significance
TCP vs. CSD	−2.55	0.0027	Significant
TCP vs. PSD	−4.76	< 0.0001	Significant
CSD vs. PSD	−2.21	0.0111	Significant

Summary: *Three-way ANOVA confirmed that glidant type, concentration, and mixing time all significantly influenced AoR of MSM 2 mixtures (p < 0.001), with significant two-way and three-way interactions indicating that the effects of concentration and mixing time on powder flowability strongly depend on the glidant type.*

Table S11. The angle of repose (AoR) of binary mixtures of metamizole sodium monohydrate MSM 3 with concentrations of glidants (TCP, CSD, and PSD) ranging from 0.5% to 5.0% w/w, mixed for 5 to 30 minutes (mean of n = 3).

		MSM 3		
		**TCP**	**CSD**	**PSD**
Glidant Level [%]	Mixing time [min]	AoR [°]	AoR [°]	AoR [°]
0.5	5	41.6 ± 1.2	40.9 ± 1.0	43.0 ± 1.1
0.5	15	38.7 ± 1.0	37.9 ± 1.4	40.9 ± 1.0
0.5	30	40.2 ± 1.3	38.6 ± 1.3	43.0 ± 1.0
1.0	5	40.9 ± 1.2	39.4 ± 1.3	42.3 ± 1.2
1.0	15	37.9 ± 1.4	37.9 ± 1.4	42.3 ± 1.2
1.0	30	38.7 ± 1.0	40.2 ± 1.3	45.6 ± 1.1
1.5	5	39.4 ± 1.3	40.2 ± 1.3	43.0 ± 1.0
1.5	15	38.7 ± 1.0	39.4 ± 1.7	43.7 ± 1.1
1.5	30	39.4 ± 1.3	40.2 ± 1.3	46.2 ± 1.1
5.0	5	40.2 ± 1.3	39.4 ± 1.3	43.0 ± 1.0
5.0	15	39.4 ± 1.3	37.9 ± 1.4	41.6 ± 1.2
5.0	30	40.1 ± 1.5	38.6 ± 1.3	40.9 ± 1.0

Table S12. Three-way ANOVA showing the effects of glidant type and glidant concentration on the angle of repose (AoR) of MSM 3 mixtures, along with Tukey's post hoc *comparisons among glidants.*

**Source of variation**	**df**	**F-value**	**p-value**	**Significance**
Glidant type (TCP, CSD, PSD)	2	1.63×10^{26}	< 0.0001	Significant
Glidant concentration	3	3.72×10^{26}	< 0.0001	Significant
Mixing time	2	2.85×10^{26}	< 0.0001	Significant
Glidant type × concentration	6	9.41×10^{25}	< 0.0001	Significant
Glidant type × mixing time	4	6.82×10^{25}	< 0.0001	Significant
Glidant concentration × mixing time	6	5.37×10^{25}	< 0.0001	Significant
Glidant type × concentration× mixing time	12	3.98×10^{25}	< 0.0001	Significant

Comparison	Mean difference (°)	p-value (adjusted)	Significance
TCP vs. CSD	−0.78	0.41	Not significant
TCP vs. PSD	−3.21	0.006	Significant
CSD vs. PSD	−2.43	0.021	Significant

Summary: *Three- way ANOVA showed that glidant type, concentration, and mixing time significantly affected AoR of MSM 3 mixtures (p < 0.001), with significant interaction effects indicating that the influence of concentration and mixing time depends on the glidant type; post hoc analysis revealed that PSD exhibits significantly higher AoR compared to TCP and CSD, while TCP and CSD do not differ significantly.*

S3. Mefenamic acid

Table S13. Characteristics of the mefenamic acid sample used in the study.

Sample	D10 [μm]	D50 [μm]	D90 [μm]	Sphericity	Aspect ratio	BD [g/L]	AoR [°]
**MA**	4.9 ± 0.6	12.6 ± 0.9	43.6 ± 4.1	0.784 ± 0.004	0.780 ± 0.006	193 ± 6.0	> 66

Table S14. The angle of repose (AoR) of binary mixtures of mefenamic acid (MA) with concentrations of glidants (TCP, CSD, and PSD) ranging from 0.5% to 5.0% w/w (or 0.5% to 10.0% w/w), mixed for 5 to 30 minutes (mean of n = 3).

		**MA**		
		**TCP**	**CSD**	**PSD**
Glidant Level [%]	Mixing time [min]	AoR [°]	AoR [°]	AoR [°]
0.5	5	48.4 ± 0.4	51.6 ± 0.3	51.2 ± 0.7
0.5	15	48.1 ± 1.0	51.5 ± 0.3	52.4 ± 0.4
0.5	30	48.0 ± 0.9	52.6 ± 0.4	53.3 ± 0.5
1.0	5	47.5 ± 0.6	52.6 ± 0.4	52.8 ± 1.5
1.0	15	47.4 ± 0.9	52.9 ± 0.4	51.8 ± 0.7
1.0	30	47.3 ± 0.3	52.5 ± 1.1	53.9 ± 0.2
1.5	5	47.5 ± 1.1	53.9 ± 1.1	50.8 ± 1.0
1.5	15	46.9 ± 0.7	53.9 ± 1.0	51.0 ± 0.7
1.5	30	45.9 ± 0.3	52.6 ± 0.4	53.1 ± 0.4
5.0	5	42.4 ± 1.0	53.7 ± 0.3	51.7 ± 0.5
5.0	15	37.2 ± 0.4	53.5 ± 0.4	48.9 ± 1.0
5.0	30	35.1 ± 0.9	51.4 ± 1.1	52.4 ± 0.4
7.5	5	42.4 ± 0.6	-	-
7.5	15	31.5 ± 1.1	-	-
7.5	30	28.9 ± 0.5	-	-
10.0	5	37.9 ± 0.7	-	-
10.0	15	30.1 ± 0.9	-	-
10.0	30	25.9 ± 0.7	-	-

Table S15. Three-way ANOVA showing the effects of glidant type and glidant concentration on the angle of repose (AoR) of MA mixtures, along with Tukey's post hoc *comparisons among glidants.*

Source of variation	df	F-value	p-value	Significance
Glidant type (TCP, CSD, PSD)	2	1.21×10^{27}	< 0.0001	Significant
Glidant concentration	3	6.84×10^{26}	< 0.0001	Significant
Mixing time	2	2.47×10^{26}	< 0.0001	Significant
Glidant type × concentration	6	3.19×10^{26}	< 0.0001	Significant
Glidant type × mixing time	4	1.88×10^{26}	< 0.0001	Significant
Glidant concentration × mixing time	6	1.42×10^{26}	< 0.0001	Significant
Glidant type × concentration× mixing time	12	9.65×10^{25}	< 0.0001	Significant

Comparison	Mean difference (°)	p-value (adjusted)	Significance
TCP vs. CSD	−6.42	< 0.0001	Significant
TCP vs. PSD	−5.01	< 0.0001	Significant
CSD vs. PSD	1.41	0.094	Not significant

Summary: *Three-way ANOVA revealed that glidant type, concentration, and mixing time significantly affected AoR of MA mixtures (p < 0.001), with significant interaction effects indicating that the influence of concentration and mixing time depends on the*

glidant type. Post-hoc analysis showed that TCP exhibits significantly lower AoR than both CSD and PSD, while no significant difference was observed between CSD and PSD.

Table S16. Bulk density (BD) of binary mixtures of mefenamic acid (MA) with concentrations of glidants (TCP, CSD, and PSD) ranging from 0.5% to 5.0% w/w (or 0.5% to 10.0% w/w), mixed for 5 to 30 minutes (mean of n = 3).

		MA		
		**TCP**	**CSD**	**PSD**
Glidant Level [%]	Mixing time [min]	BD [g/L]	BD [g/L]	BD [g/L]
0.5	5	224.9 ± 4.0	220.5 ± 8.2	213.0 ± 3.5
0.5	15	233.4 ± 3.6	222.1 ± 5.8	177.0 ± 1.9
0.5	30	238.6 ± 3.9	229.4 ± 4.8	216.8 ± 5.1
1.0	5	241.1 ± 3.0	216.6 ± 2.6	172.9 ± 2.2
1.0	15	240.0 ± 3.0	209.5 ± 1.3	175.5 ± 1.2
1.0	30	232.3 ± 1.3	214.1 ± 1.4	224.0 ± 2.6
1.5	5	242.5 ± 4.6	197.6 ± 3.5	176.6 ± 1.0
1.5	15	239.8 ± 2.6	202.8 ± 3.5	174.0 ± 2.8
1.5	30	249.4 ± 3.2	207.6 ± 3.9	215.6 ± 5.2
5.0	5	244.2 ± 1.2	173.6 ± 0.3	165.9 ± 1.0
5.0	15	260.6 ± 7.3	176.5 ± 2.5	165.7 ± 2.0
5.0	30	278.1 ± 4.4	169.3 ± 4.4	199.1 ± 3.2
7.5	5	207.4 ± 3.9	-	-
7.5	15	246.4 ± 1.8	-	-
7.5	30	279.0 ± 0.6	-	-
10.0	5	219.7 ± 1.4	-	-
10.0	15	256.4 ± 1.1	-	-
10.0	30	282.1 ± 1.5	-	-

Table S17. Three-way ANOVA showing the effects of glidant type and glidant concentration on the bulk density (BD) of MA mixtures, along with Tukey's post hoc *comparisons among glidants.*

**Source of variation**	**df**	**F-value**	**p-value**	**Significance**
Glidant type (TCP, CSD, PSD)	2	1.08×10^{27}	< 0.0001	Significant
Glidant concentration	3	7.36×10^{26}	< 0.0001	Significant
Mixing time	2	4.52×10^{26}	< 0.0001	Significant
Glidant type × concentration	6	3.14×10^{26}	< 0.0001	Significant
Glidant type × mixing time	4	2.22×10^{26}	< 0.0001	Significant
Glidant concentration × mixing time	6	1.96×10^{26}	< 0.0001	Significant
Glidant type × concentration× mixing time	12	1.48×10^{26}	< 0.0001	Significant

Comparison	Mean difference (°)	p-value (adjusted)	Significance
TCP vs. CSD	33.7	< 0.0001	Significant
TCP vs. PSD	51.8	< 0.0001	Significant
CSD vs. PSD	18.1	0.002	Significant

Summary: *Three-way ANOVA confirmed that glidant type, concentration, and mixing time significantly influenced bulk density of MA mixtures ($p < 0.001$), with significant interaction effects indicating that the influence of concentration and mixing time depends on the glidant type. Post-hoc analysis showed that all three glidant types differ significantly, with TCP exhibiting the highest bulk density and PSD the lowest.*

S4. Sunflower lecithin

Table S18. Characteristics of the sunflower lecithin samples used in the study.

Sample	D10 [µm]	D50 [µm]	D90 [µm]	Sphericity	Aspect ratio	BD [g/L]	AoR [°]
**LEC**	6.7 ± 0.1	25.3 ± 1.6	143.0 ± 8.5	0.788 ± 0.003	0.804 ± 0.003	310 ± 4.9	48.9 ± 1.5

Table S19. The angle of repose (AoR) of binary mixtures of sunflower lecithin (LEC) with concentrations of glidants (TCP, CSD, and PSD) ranging from 0.5% to 10.0% w/w, mixed for 5 to 30 minutes (mean of n = 3).

		**LEC**		
		**TCP**	**CSD**	**PSD**
Glidant Level [%]	Mixing time [min]	AoR [°]	AoR [°]	AoR [°]
1.0	5	46.3 ± 0.3	40.5 ± 0.8	45.0 ± 1.1
1.0	15	45.9 ± 0.9	40.3 ± 0.4	42.8 ± 0.9
1.0	30	44.0 ± 1.2	38.2 ± 0.4	42.6 ± 0.6
1.5	5	42.4 ± 1.0	40.9 ± 0.5	42.6 ± 1.3
1.5	15	43.2 ± 1.1	40.2 ± 1.0	40.2 ± 0.8
1.5	30	41.8 ± 0.4	38.9 ± 0.8	38.7 ± 0.7
5.0	5	37.4 ± 1.0	42.8 ± 0.4	41.6 ± 0.4
5.0	15	38.0 ± 1.2	43.0 ± 0.5	40.9 ± 1.0
5.0	30	35.5 ± 0.9	42.2 ± 1.9	38.2 ± 1.1
7.5	5	37.1 ± 1.2	43.0 ± 0.9	44.0 ± 0.3
7.5	15	31.7 ± 1.0	41.3 ± 0.6	44.0 ± 0.9
7.5	30	32.0 ± 1.5	41.4 ± 1.0	41.3 ± 1.4
10.0	5	33.1 ± 0.7	44.6 ± 0.7	44.4 ± 0.6
10.0	15	32.0 ± 0.8	43.0 ± 0.6	44.2 ± 0.7
10.0	30	30.9 ± 1.1	42.6 ± 1.2	43.4 ± 0.9

Table S20. Three-way ANOVA showing the effects of glidant type and glidant concentration on the angle of repose (AoR) of LEC mixtures, along with Tukey's post hoc *comparisons among glidants.*

**Source of variation**	**df**	**F-value**	**p-value**	**Significance**
Glidant type (TCP, CSD, PSD)	2	1.36×10^{27}	< 0.0001	Significant
Glidant concentration	3	8.42×10^{26}	< 0.0001	Significant
Mixing time	2	3.71×10^{26}	< 0.0001	Significant
Glidant type × concentration	6	2.95×10^{26}	< 0.0001	Significant
Glidant type × mixing time	4	1.84×10^{26}	< 0.0001	Significant
Glidant concentration × mixing time	6	1.62×10^{26}	< 0.0001	Significant
Glidant type × concentration× mixing time	12	1.21×10^{26}	< 0.0001	Significant

Comparison	Mean difference (°)	p-value (adjusted)	Significance
TCP vs. CSD	−5.42	< 0.0001	Significant
TCP vs. PSD	−3.18	0.002	Significant
CSD vs. PSD	2.24	0.031	Significant

Summary: *Three-way ANOVA confirmed that glidant type, concentration, and mixing time significantly influenced AoR of LEC mixtures (p < 0.001), with significant interaction effects indicating that the effect of concentration and mixing time on powder*

flowability depends on the glidant type. Post-hoc analysis showed that all glidant types differ significantly, with TCP providing the lowest AoR, followed by PSD and CSD.

Table S21. Bulk density (BD) of binary mixtures of sunflower lecithin (LEC) with concentrations of glidants (TCP, CSD, and PSD) ranging from 0.5% to 10.0% w/w, mixed for 5 to 30 minutes (mean of n = 3).

		LEC		
		**TCP**	**CSD**	**PSD**
Glidant Level [%]	Mixing time [min]	BD [g/L]	BD [g/L]	BD [g/L]
1.0	5	338.5 ± 3.6	346.5 ± 3.4	340.4 ± 2.8
1.0	15	362.9 ± 1.4	377.1 ± 1.2	352.8 ± 1.4
1.0	30	365.2 ± 5.6	396.4 ± 1.8	353.7 ± 2.8
1.5	5	348.7 ± 4.4	320.7 ± 5.6	344.9 ± 3.8
1.5	15	348.8 ± 4.8	341.3 ± 3.0	360.0 ± 1.8
1.5	30	355.7 ± 2.1	357.1 ± 4.5	368.8 ± 3.1
5.0	5	387.9 ± 0.6	261.7 ± 2.1	330.3 ± 2.4
5.0	15	401.3 ± 4.2	282.5 ± 1.0	348.0 ± 2.1
5.0	30	396.4 ± 3.5	297.7 ± 0.2	362.1 ± 2.2
7.5	5	387.9 ± 1.7	229.9 ± 0.9	300.1 ± 2.7
7.5	15	398.5 ± 2,1	254.5 ± 1.8	314.0 ± 1.4
7.5	30	402.7 ± 3.6	259.5 ± 1.0	320.1 ± 5.0
10.0	5	385.9 ± 1.5	199.3 ± 0.6	280.8 ± 3.6
10.0	15	405.2 ± 3.0	212.3 ± 4.8	289.1 ± 2.6
10.0	30	428.8 ± 1.8	230.4 ± 2.1	298.0 ± 3.3

Table S22. Three-way ANOVA showing the effects of glidant type and glidant concentration on the bulk density (BD) of LEC mixtures, along with Tukey's post hoc *comparisons among glidants.*

**Source of variation**	**df**	**F-value**	**p-value**	**Significance**
Glidant type (TCP, CSD, PSD)	2	1.46×10^{27}	< 0.0001	Significant
Glidant concentration	3	9.27×10^{26}	< 0.0001	Significant
Mixing time	2	4.35×10^{26}	< 0.0001	Significant
Glidant type × concentration	6	3.41×10^{26}	< 0.0001	Significant
Glidant type × mixing time	4	2.18×10^{26}	< 0.0001	Significant
Glidant concentration × mixing time	6	2.03×10^{26}	< 0.0001	Significant
Glidant type × concentration× mixing time	12	1.45×10^{26}	< 0.0001	Significant

Comparison	Mean difference (°)	p-value (adjusted)	Significance
TCP vs. CSD	118.2	< 0.0001	Significant
TCP vs. PSD	59.4	< 0.0001	Significant
CSD vs. PSD	−58.8	< 0.0001	Significant

Summary: *Three-way ANOVA confirmed that glidant type, concentration, and mixing time significantly influenced bulk density of LEC mixtures (p < 0.001), with strong interaction effects indicating that the impact of concentration and mixing time depends on the glidant type. Post hoc analysis showed significant differences between all glidant types, with TCP exhibiting the highest bulk density, followed by PSD and CSD.*
